# Sex Differences in Rehabilitation Outcomes After Stroke: A Single-Center Retrospective Observational Study

**DOI:** 10.3390/jcm15218660

**Published:** 2026-11-06

**Authors:** Michał Małek, Anna Hozakowska, Zbigniew Guzek, Małgorzata Stefańska, Joanna Kowalska

**Affiliations:** 1 Department of Physiotherapy, University of Zielona Góra, 65-046 Zielona Góra, Poland; m.malek@inz.uz.zgora.pl (M.M.); a.hozakowska@inz.uz.zgora.pl (A.H.); 2 Department of Neurological Rehabilitation, University Hospital in Zielona Góra, 65-046 Zielona Góra, Poland; guzek@o2.pl; 3 Faculty of Physiotherapy, Wroclaw University of Health and Sport Sciences, 50-367 Wrocław, Poland; malgorzata.stefanska@awf.wroc.pl

**Keywords:** stroke, sex, rehabilitation outcome, post-stroke rehabilitation, functional status, activity of daily living

## Abstract

**Background/Objectives**: The findings of studies that assess post-stroke rehabilitation outcomes according to sex remain inconclusive. This study aimed to assess the rehabilitation outcomes among patients after a first stroke according to sex and identify the factors related to functional status and independence in performing basic activities of daily living at discharge. **Methods**: The data from 527 patients, aged 69.1 ± 11.8 years, comprising 244 women and 283 men, were analyzed. The Mini Mental State Examination (MMSE), Barthel Index (BI), Trunk Control Test (TCT), Timed Up and Go Test (TUG), and Berg Balance Scale (BBS) were used at two measurement points: upon admission to the ward (T1) and at discharge (T2). **Results**: No significant differences between women and men at T1 were found. At T2, statistically significant differences in the length of stay in the ward, MMSE, BBS, TCT, TUG, and BI were observed, but the reported effect sizes were generally small. At T2, men had better rehabilitation outcomes, but they stayed in the rehabilitation ward significantly longer than women. The analysis revealed no significant effect of the time point of measurement (T1 vs. T2) and sex interaction on the test results. Greater independence in basic activities of daily living at discharge (BI > 15 at T2) depended on sex, age, MMSE, and TUG at T1. **Conclusions**: The results obtained do not provide conclusive evidence for differences in rehabilitation outcomes based on sex. Further research on the factors and mechanisms involved in stroke rehabilitation outcomes in relation to sex is needed.

## 1. Introduction

In the current era of medicine, it has become crucial to take sex into account in the study of post-stroke patients. Differences in pathophysiology, acute-phase treatment response, and subsequent post-stroke rehabilitation require careful clinical analysis [1].

Contemporary studies have highlighted the increasing significance of dimorphic differences in the clinical course and consequences of stroke. Alongside age and comorbidities, these differences appear to influence the progression of symptoms, the patient’s clinical picture in the acute phase, and the subsequent rate and extent of neurological and functional recovery [2].

Consequently, increasing attention is being devoted to factors that can modify rehabilitation outcomes, with biological sex being a subject of growing interest [2,3,4].

Research has indicated that women after stroke onset are older than men and more frequently burdened with multiple comorbidities and specific risk factors, such as pregnancy complications; hormone replacement therapy, including oral contraceptives; and longer lives, contributing to a higher occurrence of depression, diabetes, and atrial fibrillation. The loss of the cardioprotective effect of estrogens after menopause also appears to be significant, resulting in women being more frequently affected by strokes with a more severe course [5,6].

In men, the earlier occurrence of vascular events is more commonly observed, which is associated with a different risk factor profile—hypertension, dyslipidemia, and lifestyle factors predominate in these cases [2,5].

Despite growing awareness of sex differences in medicine, the findings of studies assessing post-stroke rehabilitation outcomes by sex remain inconclusive [5,7,8].

While most research has indicated that women have poorer functional outcomes after stroke compared to men, some studies have described favorable recovery patterns, particularly in specific age groups or with specific treatments [5,9,10].

Discrepancies in existing research findings stem from a variety of factors, including biopsychosocial factors, significant heterogeneity in research methodologies, patient groups and populations, measurement tools, and follow-up duration [11,12].

This has prompted ongoing updates to the research with more detailed analyses [5], taking into account, among other things, treatment methods and the rehabilitation process itself [9].

This is also particularly important in the context of greater awareness among both women and men of stroke itself, including its prevention, symptoms, treatment options and methods, the role of rehabilitation in recovery and regaining independence, and access to treatment and rehabilitation, as well as their continuation after a stroke [3,13].

Understanding these relationships is essential for optimizing treatment, recovery, and therapy processes within a holistic model of care for stroke patients [7,14].

Pursuing the greatest possible effectiveness in rehabilitation, initially in the neurology ward and then in post-stroke rehabilitation wards, and rapidly achieving an optimal functional state may significantly reduce patients’ hospital stays after stroke. This enables them to return home and/or resume family and working life as soon as possible [7,15]. This is particularly important when considering the patient’s improvement and regained independence in activities of daily living, an area that has not yet been studied enough. Sex-specific rehabilitation strategies in stroke recovery remain insufficiently explored.

Therefore, the aim of the study was to retrospectively assess rehabilitation outcomes among patients after a first stroke according to sex and identify the factors related to functional status and independence in performing basic activities of daily living following discharge from post-stroke rehabilitation wards.

Updating the research on post-stroke rehabilitation that considers sex appears to be essential from both a scientific perspective and practical implications. Analyzing these results could help to improve the planning of therapeutic strategies, provide a better understanding of the differences in the process of regaining self-sufficiency and independence, and enable them to be adapted them to the specific needs of each patient group.

## 2. Materials and Methods

### 2.1. Studied Group

A retrospective observational study was conducted at the Neurological Rehabilitation Ward within the Department of Rehabilitation at the University Hospital in Zielona Góra, Poland, and received a positive opinion from the Ethics Committee of Wroclaw University Health and Sport Sciences in Wroclaw, Poland (reference no 23/2024). The completed STROBE checklist is available as Supplementary Material. The study was conducted in accordance with the Declaration of Helsinki.

Data were collected from the medical records of patients consecutively admitted from the neurological ward to the post-stroke rehabilitation ward who met the following inclusion criteria: first-ever stroke, with admission to the ward between January 2020 and December 2025. Patients with diagnoses other than stroke and those lacking documented results of cognitive and functional status assessments were excluded from the analysis. The complete-case analysis was used.

**Figure 1:**
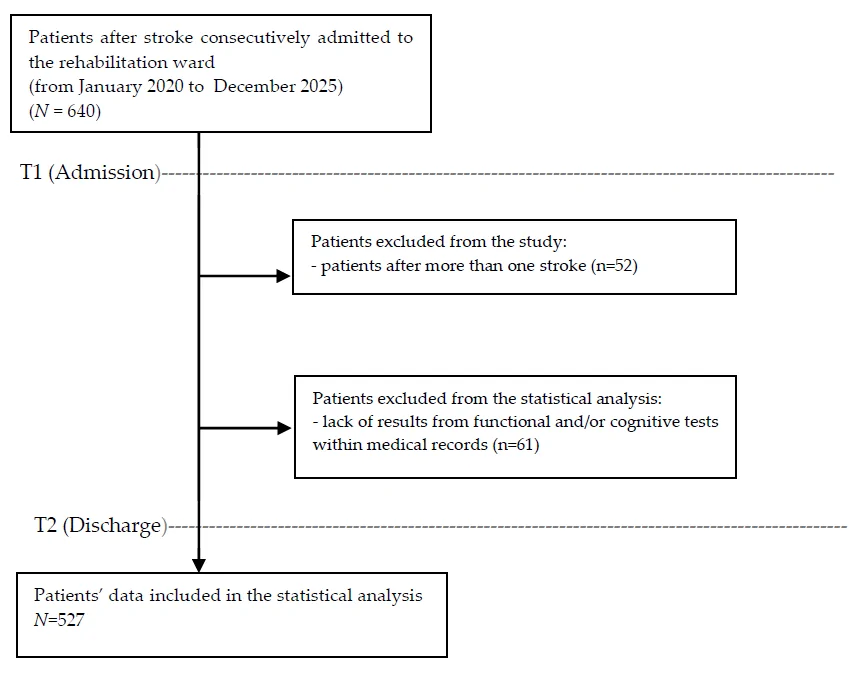
Data collection for statistical analysis.

Finally, data from 527 patients—aged 69.1 ± 11.8 years, comprising 244 women (46.3%) and 283 men (53.7%)—were subjected to final analysis (Figure 1).

An ischemic stroke mechanism and a lesion location in the right cerebral hemisphere dominated the clinical characteristics of the group. Men were significantly younger than women. Otherwise, no statistically significant differences were observed between the groups of women and men (Table 1).

### 2.2. Measurement Tools

Based on medical records, the following data were collected: sociodemographic information (age, sex, marital status); stroke-related data (type of stroke, affected hemisphere, presence of aphasia, number of days from stroke to admission to the post-stroke rehabilitation ward, length of stay in the ward); and the results of tests standardly performed in the ward—Mini Mental State Examination (MMSE), Barthel Index (BI), Trunk Control Test (TCT), Timed Up and Go Test (TUG), and Berg Balance Scale (BBS).

The Polish MMSE, as adapted by Stańczak, is used for the screening assessment of patients’ cognitive status and the suspicion of cognitive impairment and dementia. Patients can achieve up to 30 points. A score of 30–27 points indicates normal cognitive function and an absence of cognitive impairment, while a score below 24 points suggests the suspicion of dementia [16].

The BI assesses functional status and the ability to perform basic activities of daily living. Higher scores indicate a better functional status and a greater ability to function independently. Participants can achieve up to 20 points. Scores of 0–7 indicate severe functional status, 8–15 moderate functional status, and 16–20 mild functional status [17,18].

The TCT is used to assess in-bed mobility and postural control while in a sitting position. It consists of four movement tasks. Each task is rated on a three-tier scale: 0—unable to perform the task without assistance; 12—able to perform the task but in an abnormal way; and 25—able to perform the task normally. Patients can obtain a maximum of 100 points [19].

The TUG is a screening tool used to test basic mobility skills: postural control, balance, and walking ability. The test records the time required, in seconds, for a participant to rise from a chair, walk three meters, turn around, and return to a seated position. Patients can use orthopedic aids, such as a cane or walker. The shorter the time, the lower the risk of falling [20]. Due to the rapid changes and generally poorer initial conditions of patients after stroke, a nominal scale was adopted (inability to perform/test performed), which indicates improvement in a patient’s mobility [21].

The BBS is designed to evaluate both static and dynamic balance. It comprises 14 movement tasks, each scored on a scale 0–4 points, with a maximum total score of 56. Higher scores indicate better balance and a lower risk of falling. Specifically, scores from 0 to 20 indicate a high risk of falling, 21 to 40 suggest a moderate risk, and scores 41 to 56 represent a low risk [22].

Assessments were performed at two measurement time points: upon admission to the ward (T1) and at the time of the patient’s discharge (T2). The same assessors performed the T1 and T2 assessments.

During their stay on the ward, patients regularly followed an individualized rehabilitation program tailored to their psychophysical capabilities. This program was specified in terms of frequency and duration (averaging 120 min per day, five days a week from Monday to Friday, with 45 min on Saturdays). This rehabilitation program consisted of 90 min individual sessions with a physiotherapist, focusing on exercises to increase muscle strength in the trunk and upper and lower limbs and to improve limb function. It also included balance exercises for both low and high positions.

The 30 min gait training sessions included learning to walk on a both flat and uneven surfaces and up and down stairs while further improving walking techniques. During rehabilitation, methods such as Proprioceptive Neuromuscular Facilitation (PNF) and NDT-Bobath were employed. Patients also participated in occupational therapy and, if they had aphasia, speech therapy, averaging three 45 min sessions per week. Prior to admission to the post-stroke rehabilitation ward, all patients had undergone rehabilitation in the neurology ward following their stroke. The frequency and duration of rehabilitation and other therapies were comparable between women and men.

### 2.3. Statistical Analysis

Due to the ordinal nature of most variables and the absence of a normal distribution, the medians and interquartile ranges (IQRs) were calculated. For nominal variables, numbers and percentages were calculated.

The Mann–Whitney U test was used to check the significance of differences between the groups of women and men for ordinal and quantitative variables, and the chi-square test was used for qualitative variables—alternatively, Fisher’s exact test was used if Cochran’s rule was not met. The effect size for the Mann–Whitney U test was estimated using the eta-squared statistic, which was then transformed into Cohen’s d [23]. The effect size for 2 × 2 tables was determined using the Phi coefficient (φ), whereas for larger tables, Cramer’s V was used (0.1 to 0.3—weak effect; 0.3 to 0.5—moderate effect; ≥0.5—strong effect). To determine the effects of the time point of measurement, sex, and the sex × time point of measurement interaction, a generalized linear model in the form of generalized estimating equations (GEEs) was used.

Multiple regression models were estimated, with BI at T2 used as the dependent variable.

Logistic regression models were estimated to identify the predictors of patients’ independence in performing basic activities of daily living at discharge from the ward. The dependent variable in the models was the BI at T2, which was classified into two categories: BI > 15 points at T2, indicating a better functional status and the ability to perform most basic activities of daily living independently; and BI ≤ 15 points at T2, indicating the need for assistance from others. This division was based on the interpretation of the results of the 0–20-point BIs and analyses of other studies (the cutoff BI > 15 for 0–20 points corresponds to the cutoff BI > 75 for 0–100 points) [24,25,26].

Sensitivity was assessed using Cook’s and Mahalanobis distances. No observations were found to exceed the critical threshold for Cook’s distance, nor did they exceed 3 standard deviations for Mahalanobis distance.

Statistical significance was set at *p* < 0.05. The complete-case analysis was used. The analysis was performed using Statistica 14.1 and PQStat 1.8.4, IBM SPSS Statistic 32.0.0.0, as well as with online statistical calculators (https://www.psychometrica.de/effect_size.html; accessed on 19 July 2026).

## 3. Results

An analysis of the results showed no significant differences between the female and male groups at T1. At T2; however, the male group showed significantly higher MMSE, BBS, and BI scores.

Scores indicating no suspicion of dementia (MMSE at T2) were observed in 52% of women and 66% of men. Greater independence (BI >15) at T2 was observed in 49% of women and 62% of men. A low risk of falling (BBS > 40 at T2) was observed in 52% of women and 65% of men.

Statistically significant differences were also observed in the TCT and TUG scores at T2. Significantly longer lengths of stays in the post-stroke rehabilitation ward were observed in men (Table 2).

However, it is worth remembering that the higher age of women in this study represented a potential confounding factor, and group differences at discharge cannot be fully attributed to sex alone.

At T1, only 170 patients were able to perform the TUG; at T2, this number increased to 380 patients. Table 2 presents the TUG results (in seconds) for only those who completed the assessment. To provide a comprehensive overview of the changes, an additional analysis was conducted in which the TUG results were converted into a nominal scale, where zero indicated an inability to complete the TUG and one indicated its successful completion. It was observed that, at T1, only about 30% of the participants were able to complete the test, whereas at T2, more than 70% performed it (Table 3).

To examine the influence of sex on the test results, a generalized linear model in the form of generalized estimating equations (GEEs) was applied. The analysis revealed no significant effect of the interaction between the time point when measurement was taken (T1 vs. T2) and sex on the test results. However, both the time point of measurement (T1 vs. T2) and sex were found to have a significant effect on the MMSE, BBS, and BI scores (Table 4).

The multiple regression model estimated for the entire study group (*n* = 527) revealed significant effects of age, MMSE at T1, and TUG at T1 on a patient’s functional status at discharge from the ward (BI at T2) (Table 5).

The analysis showed that, of the 527 stroke patients who began rehabilitation in the post-stroke rehabilitation ward, only 1% had a functional status that allowed them to perform the most basic activities of daily living independently (BI > 15 at T1). At T2, this status was confirmed in 56% of all patients (n = 295), including 41% of women (n = 120) and 59% of men (n = 175).

To predict functional status and independence at discharge from the ward (BI at T2), patients were divided into two groups: patients with a BI > 15 points at T2, indicating a better functional status and the ability to perform most basic activities of daily living independently; and patients with a BI ≤ 15 points at T2, indicating a poorer functional status and the need for assistance from others. A logistic regression model was estimated. The dependent variable was BI > 15 at T2.

The model showed significant positive odds ratios for sex, age, MMSE at T1, and TUG at T1. However, no significant effects were found for the stroke type, affected hemisphere, presence of aphasia, time from stroke to admission to the rehabilitation ward, and length of stay in the ward (Table 6).

## 4. Discussion

The study’s results have revealed significant insights into the dynamics of changes in the psychophysical states for stroke patients undergoing comprehensive post-stroke rehabilitation. The study has shown potential sex differences among the prognostic factors known at the time of admission to the post-stroke rehabilitation ward (T1).

At the time of admission to the ward (T1), only 1% of the 527 patients had a functional status that allowed greater independence in performing basic activities of daily living (BI > 15 at T1), whereas at discharge (T2), this percentage increased to 56%. This indicates an improvement in the patient’s functional status during post-stroke rehabilitation. However, it should be noted that improvements observed between T1 and T2 may be due to spontaneous neurological or natural recovery, rehabilitation, or a combination of these factors.

At T1, no significant differences were observed between the women’s and men’s groups. However, at T2, men achieved significantly better results in cognitive function, as well as in the other tests concerning balance, mobility and gait, and independence in performing basic activities of daily living. Analysis of TUG performance also showed that significantly more men completed the test at T2.

These observations are consistent with reports from other researchers. In one study, Xu et al. found that, despite better survival rates, women had poorer functional outcomes at discharge than men [4]. Fleming et al. also pointed out that, compared to men, women had a higher risk of stroke with poorer functional outcomes and higher disability rates [27]. Conversely, Irie et al. demonstrated that women who survived a stroke were at greater risk of poorer functional outcomes within five years of stroke than men [28]. However, Chen et al. found that three months after stroke, outcomes improved similarly for both sexes, but this pattern of recovery differed by sex [11]. Cocco et al. reported that, at both admission and discharge from the ward, women had a significantly worse functional status than men [7]. On the other hand, Poggesi et al. found no differences in the functional status of women and men at the time of admission to the ward [5]. At discharge, however, the authors reported findings that differed from ours: men and women continued to exhibit similar functional and clinical status. At the same time, the authors also noted that the potential for recovery appeared to be greater in older women than in older men [5].

In contrast, Hay et al. found that women performed as well as if not better than men in most activities of daily living [29]. A scoping review by Rahmaniharedasht et al. also noted that differences in rehabilitation outcomes between women and men depended on the types of technology used. For example, women showed superior performance in robotic gait training, functional electrical stimulation, hand function using soft robotic gloves, and virtual-reality-based balance recovery. In this analysis, younger women achieved better results than men in motor tasks, and this trend was reversed with age [10].

The observed differences in post-stroke rehabilitation outcomes are often attributed to the fact that women tend to be older when they have a stroke, have poorer overall health before stroke, and are more likely to live alone. This may negatively affect the pace of their recovery [5,6]. Additionally, Mavridis et al. found that women received less comprehensive treatment in the stroke ward as older adults when compared to what younger adults receive [9]. It is also worth noting that in this study, men spent significantly more time in the ward than women. This difference in the length of stay may have contributed to better outcomes in men by allowing them more time for neuroplasticity and physical therapy (neuroplasticity and functional recovery). While this is a plausible hypothesis, it cannot be confirmed based on the present observational analysis. Nevertheless, most researchers have indicated that a shorter length of stay is associated with better functional outcomes at discharge, and that patients with a higher degree of disability require longer hospital stays [30,31]. Meanwhile, in the present study, women had a shorter length of stay in the ward despite having poorer functional status at discharge compared to men. In our opinion, this finding requires confirmation, but it suggests that therapeutic interventions in the rehabilitation process may need to be modified to take into account the needs of both sexes.

Further analyses conducted using generalized estimating equations (GEEs) revealed statistically significant main effects for both sex and the measurement time point (T1 vs. T2). Significant differences were found between men and women in the MMSE, BBS, and BI scores (in the case of BI, this aligns with the previously described clinical significance), with significant improvements in the MMSE, TCT, TUG, and BI scores between T1 and T2. However, no significant sex × measurement time point (T1 vs. T2) interaction was observed, indicating no evidence of a differential responses to rehabilitation between men and women. This suggests that the nature of these changes was similar in both groups regardless of any differences in the overall scores achieved. These results suggest that further research focusing on sex is required.

Nevertheless, baseline clinical differences may still influence subsequent outcomes, even if they do not reach conventional statistical significance. Additionally, while several sex differences reached statistical significance at T2, the reported effect sizes were generally small. However, statistical significance should not automatically be equated with clinically significant differences.

In the present study, the differences within the individual groups significantly exceeded the established minimal clinically important difference (MCID) values for all test results. However, the differences in the dynamics of change between these groups (women and men) were clinically significant only in the case of BI and TCT. According to Hsieh et al., in the 20-point version of the BI, MCID was approximately two points (1.85) [26]. For the TCT according to Nozoe et al., MCID was stated to be 13 points, meaning that a change in the TCT of at least 13 points reflects a genuine improvement in functioning which is noticeable to the patient [32].

The results obtained in this study exceed these thresholds. Men showed greater clinical improvement in their MMSE and BI scores than women. However, better clinical improvement in the TCT was found in women.

These changes signify a real and noticeable improvement in the patient’s daily functioning. However, our results should be interpreted with caution due to certain limitations and the further research is required.

The regression analysis did not confirm that sex was a predictor of functional status at T2. The regression model identified younger age, cognitive status at T1, and the ability to complete the TUG at T1 as predictors of better functional status (BI at T2) and greater independence in performing basic activities of daily living at discharge (BI > 15 at T2). Additionally, male sex was a predictor of greater independence in performing basic activities of daily living at discharge (BI > 15 at T2). In the logistic regression model, male sex was associated with nearly 1.7-fold higher odds of achieving functional independence (OR = 1.72; 95% CI: 1.08–2.73).

Similar results were obtained by Mayridis et al., who demonstrated that sex and age were key factors determining the final functional status [9]. The impact of age on post-stroke prognosis was directly linked to the physiological depletion of neural reserves and higher comorbidity in older adults, resulting in poorer outcomes for elderly patients, as described by Abdu and Seyouma [2].

Cognitive status and mobility upon admission to the post-stroke rehabilitation ward were significant factors in predicting final functional outcomes at discharge. Patients with even a low degree of mobility upon admission to post-stroke rehabilitation ward (e.g., moving within the confines of their bed or moving with assistance) were more likely to regain independence in performing activities of daily living at the time of discharge.

However, research has indicated that the cognitive status at the time of starting the rehabilitation process, rather than functional status, is the primary predictor of functional status at discharge among various patient groups, including stroke patients [33,34]. Patients without cognitive deficits or symptoms of dementia had a better understanding of therapeutic instructions, demonstrated a higher level of motivation, and consolidated new movement patterns more effectively during the motor learning process in rehabilitation. Therefore, this result is not surprising, as it confirms previous research findings. However, it merely indicates that both the patient’s cognitive function and mobility (including gait and balance) are factors that may influence post-stroke outcomes at discharge [35].

Additionally, Sawyer et al. emphasized that patients with cognitive impairment exhibited greater disability and lower BI scores, particularly following a hemorrhagic stroke [36].

These results may have practical implications. Although many factors are associated with post-stroke rehabilitation outcomes, these results may help suggest which factors (among those known in the first days after admission to the post-stroke rehabilitation ward) the therapeutic team should consider when predicting the chances of regaining independence. This could facilitate optimal planning of the rehabilitation process, albeit with a great deal of caution.

The authors acknowledge that sex differences have multifactorial causes and may stem from various biological and psychological factors. The present study only considered factors that were known at the start of rehabilitation and at the time of admission to the post-stroke rehabilitation ward. Data were collected from medical records, which limited the number of factors included in the analysis. This selection could substantially influence the observed sex differences. Among other things, the MMSE is a screening tool and does not constitute a medical diagnosis. Factors such as the treatment modality, baseline stroke severity, premorbid functional status, affected brain region, comorbidities (e.g., depression, atrial fibrillation), presence of spasticity, hemispatial neglect, pusher syndrome, associated reactions, and social support were not taken into account. As all of these factors may differ by sex and could be related to the rehabilitation outcomes, the observed association between sex and BI results at discharge cannot be interpreted as an independent biological effect of sex. The studied groups may not be representative of the entire population of patients after stroke due to the study being conducted in a single-center and the inclusion of patients transferred directly from the neurological ward. Consequently, the generalizability of the findings may be limited, and the results should be replicated in multi-center populations.

Additionally, the results are restricted to discharge status and cannot be generalized to long-term community-based prognoses.

The literature review, and the trends presented in this article, highlight the need for further research into the factors and mechanisms involved in the post-stroke rehabilitation process, particularly with regard to sex. This is important, as the guidelines for post-stroke rehabilitation often remain the same for all patients.

## 5. Conclusions

In the study group of stroke patients, despite no differences at the time of admission to the post-stroke rehabilitation ward, significant differences were observed in the length of stay, functional and cognitive status, and mobility at discharge, depending on sex. Men had better discharge outcomes, particularly in terms of functional status, but stayed in the ward significantly longer than women. Although several sex differences reached statistical significance at discharge, the reported effect sizes were generally small.

A younger age, better cognitive status, and a higher mobility level at admission predicted a better functional status at discharge.

Greater independence in basic activities of daily living at discharge depended on sex, age, cognitive status, and mobility level at the time of admission.

Due to the limited number of factors analyzed and the presence of confounding variables, the obtained results do not conclusively demonstrate differences in rehabilitation outcomes based on sex. Further research is needed into the factors and mechanisms involved in the stroke rehabilitation process that takes into account the needs and capabilities of both sexes.

## Figures and Tables

**Table 1:** Characteristics of the study group (*n* = 527).

		Women *n* = 244		Men *n* = 283		Mann–Whitney U Test	Effect Size
Parameters		Median	IQR	Median	IQR	*p*	Cohen’s d
Age [years]		72	16	68	14	<0.0001 *	0.37
		*n*	%	n	%	Chi-square /Fisher	φ /Cramer’s V
Marital status	Married	128	52.46	138	48.76	0.5372	0.06
	Widow(er)	72	29.51	83	29.33		
	Divorced	6	2.46	5	1.77		
	Single	38	15.57	57	20.14		
Type of stroke	Ischemic	216	88.52	250	88.34	0.9471	<0.01
	Hemorrhagic	28	11.48	33	11.66		
Affected hemisphere	Left	122	50.00	123	43.46	0.1335	0.07
	Right	122	50.00	160	56.54		
Aphasia at T1	No	178	72.95	224	79.15	0.0952	0.07
	Yes	66	27.05	59	20.85		

T1—first measurement; IQR—interquartile range; * *p* < 0.05.

**Table 2:** The examined parameters at T1 and T2, depending on sex.

	Women *n* = 244		Men *n* = 283		Mann–Whitney U test		Cohen’s d
Parameters	Median	IQR	Median	IQR	U	*p*	
MMSE (T1)	22	8	23	9	20,619	0.0858	0.74
MMSE (T2)	24	7	26	6	19,530.5	0.0102 *	0.81
TCT (T1)	87	52	100	51	32,090	0.1623	0.12
TCT (T2)	100	13	100	0	29,943	0.0086 *	0.23
TUG (T1) [s]	15	7	14	8	34,050.5	0.4125	0.07
TUG (T2) [s]	10	15	12	10	29,691	0.0055 *	0.24
BBS (T1)	15	30	22	29	32,274.5	0.1966	0.11
BBS (T2)	41.5	38.5	47	18	29,370	0.0031 *	0.26
BI (T1)	4	7.5	5	9	33,178	0.4395	0.07
BI (T2)	15	13	17	8	30,354.5	0.0167 *	0.21
Time from stroke [days]	12	7	11	6	32,939	0.3627	0.08
Length of stay in the ward [days]	52.5	56	63	67	31,063	0.0470 *	0.17

T1—first measurement; T2—second measurement; MMSE—Mini Mental State Examination; BI—Barthel Index; TCT—Trunk Control Test; TUG—Timed Up and Go Test; BBS—Berg Balance Scale; IQR—interquartile range; * *p* < 0.05.

**Table 3:** TUG results from the groups of women and men.

		All		Women		Men		Chi-Square	
		n	%	n	%	n	%		*p*
TUG (T1)	0	357	67.7	168	68.9	189	66.8	0.07	0.7958
	1	170	32.3	76	31.1	94	33.2		
TUG (T2)	0	147	27.9	85	34.8	62	21.9	10.89	0.0009 *
	1	380	72.1	159	65.2	221	78.1		

T1—first measurement; T2—second measurement; TUG—Timed Up and Go Test; 0—inability to complete; 1—test completed; * *p* < 0.05.

**Table 4:** Influence of the measurement time points (T1 vs. T2), sex, and their interaction—the time point of measurement (T1 vs. T2) x sex—on the MMSE, TCT, TUG, BBS, and BI scores.

Tests	Variable	Chi-Squared Wald Test	df	*p*
MMSE	sex (W vs. M)	5.10	1	0.024 *
	time point of measurement (T1 vs. T2)	57.55	1	<0.001 *
	sex x time point of measurement	1.34	1	0.248
TCT	sex (W vs. M)	5.67	1	0.17
	time point of measurement (T1 vs. T2)	23.52	1	<0.001 *
	sex x time point of measurement	0.28	1	0.595
TUG	sex (W vs. M)	0.73	1	0.393
	time point of measurement (T1 vs. T2)	51.55	1	<0.001 *
	sex x time point of measurement	0.32	1	0.57
BBS	sex (W vs. M)	4.56	1	0.033 *
	time point of measurement (T1 vs. T2)	197.37	1	<0.001 *
	sex x time point of measurement	0.03	1	0.861
BI	sex (W vs. M)	5.89	1	0.015 *
	time point of measurement (T1 vs. T2)	240.04	1	<0.001 *
	sex x time point of measurement	1.30	1	0.255

W—women; M—men; T1—first measurement; T2—second measurement; BI—Barthel Index; MMSE—Mini Mental State Examination; TUG—Timed Up and Go Test; * *p* < 0.05.

**Table 5:** Predictors of functional status at discharge (BI at T2) for the full group of stroke patients.

Dependent Variable: BI at T2	Coefficient b	Error b	−95% CI	+95% CI	*p*
Sex (women—1; men—2)	0.12	0.99	−1.82	2.07	0.9000
Age [years]	−0.09	0.02	−0.13	−0.05	<0.0001 *
Type of stroke (ischemic—1; hemorrhagic—2)	1.03	0.75	−0.46	2.51	0.1743
Affected hemisphere (left—1; right—2)	−0.64	0.58	−1.78	0.49	0.2679
Aphasia at T1 (no—0; yes—1)	−0.74	0.75	−2.21	0.72	0.3181
MMSE (T1)	0.31	0.04	0.22	0.39	<0.0001 *
TUG (T1) (inability to complete—0; test completed—1)	4.33	0.56	3.22	5.44	<0.0001 *
Time from stroke [days]	−0.01	0.04	−0.10	0.08	0.8112
Length of stay in the ward [days]	−0.03	0.02	−0.07	0.02	0.2213
interaction: sex x length of stay in the ward [days]	0.02	0.01	−0.01	0.04	0.2927
R2 corrected	0.33				
Standard error of estimation	4.94				
F	21.98				
*p*-Value	<0.0001 *				

T1—first measurement; T2—second measurement; BI—Barthel Index; MMSE—Mini Mental State Examination; TUG—Timed Up and Go Test * *p* < 0.05.

**Table 6:** Logistic regression model identifying the significant predictors of patient independence at discharge (BI > 15 at T2).

Dependent Variable: BI > 15 at T2	Coefficient b	Error b	−95% CI	+95% CI	Stat. Wald	*p*	OR	−95% CI	+95% CI
Sex (women—1; men—2)	0.54	0.24	0.08	1.01	5.30	0.0214 *	1.72	1.08	2.73
Age [years]	−0.03	0.01	−0.05	−0.01	9.08	0.0026 *	0.97	0.95	0.99
Type of stroke (ischemic—1; hemorrhagic—2)	0.30	0.36	−0.41	1.00	0.67	0.4127	1.34	0.66	2.72
Affected hemisphere (left—1; right—2)	−0.30	0.28	−0.85	0.25	1.13	0.2873	0.74	0.43	1.29
Aphasia (T1)	−0.38	0.35	−1.06	0.29	1.23	0.2676	0.68	0.35	1.34
MMSE (T1)	0.09	0.02	0.05	0.13	19.80	<0.0001 *	1.10	1.05	1.14
TUG (T1) (inability to complete—0; test completed—1)	1.85	0.30	1.25	2.44	37.03	<0.0001 *	6.33	3.49	11.47
Time from stroke [days]	0.00	0.02	−0.04	0.04	0.00	0.9721	1.00	0.96	1.04
Length of stay in the ward [days]	0.00	0.00	−0.01	0.00	0.60	0.4380	1.00	0.99	1.00
R2 (Nagelkerke)	0.34								
Chi-square	122.22								
*p*-Value	<0.0001 *								

T1—first measurement; T2—second measurement; BI—Barthel Index; MMSE—Mini Mental State Examination; TUG—Timed Up and Go Test; CI—confidence interval; * *p* < 0.05.

## Data Availability

The data presented in this study are available from the corresponding author upon request.

## Supplementary Materials

The following supporting information can be downloaded at: https://www.mdpi.com/article/10.3390/jcm15218660/s1: STROBE Statement Checklist.
